# Analysis of epidemiology, etiology and injury patterns in 2,179 digit amputations

**DOI:** 10.1038/s41598-025-18983-y

**Published:** 2025-09-23

**Authors:** Maximilian Mayrhofer-Schmid, Martin Aman, Annette Stolle, Julia Glaser, Adriana C. Panayi, Anne-Sophie Rasner, Kyle R. Eberlin, Leila Harhaus, Arne H. Boecker

**Affiliations:** 1 Department of Hand, Plastic, and Reconstructive Surgery, BG Klinik Ludwigshafen, Burn Center at Heidelberg University, Ludwigshafen, Germany; 2 Department of Hand Surgery, Peripheral Nerve Surgery and Rehabilitation, BG Klinik Ludwigshafen, Ludwigshafen, Germany; 3 Department of Hand-, Replantation-, and Microsurgery, BG Klinikum Unfallkrankenhaus Berlin and Chair of Hand-, Replantation-, and Microsurgery at the Charité Universitätsmedizin Berlin, Berlin, Germany; 4 Department of Oral and Maxillofacial Surgery, Charité - Universitätsmedizin Berlin, Corporate Member of Freie Universität Berlin, Humboldt- Universität zu Berlin, Berlin, Germany; 5 Division of Plastic and Reconstructive Surgery, Department of General Surgery, Massachusetts General Hospital, Harvard Medical School, Boston, MA USA; 6 BG Trauma Center Ludwigshafen, Ludwig-Guttmann-Str. 13, 67071 Ludwigshafen, Germany

**Keywords:** Amputation, Trauma, Hand surgery, Epidemiology, Etiology, Trauma, Epidemiology

## Abstract

As digit amputations can profoundly affect hand function and quality of life, insight into their anatomical distribution, etiology, and epidemiology is fundamental to improving treatment and prevention. This retrospective study investigates 2,179 digit amputations in 1,768 patients treated between April 2005 and December 2021 at a German Level I trauma center, excluding successful replantations. The cohort was predominantly male (89.1%) with a median age of 49 years (IQR: 34–61) and age peaks at 20–30 and 40–60 years. Occupational injuries accounted for 38.7% of cases, more frequent among males and those under 40. Temporal trends showed seasonal peaks in July and September and increased incidence on Fridays and Saturdays. Sharp injuries were the leading cause, followed by blunt trauma and avulsion. The index finger was most frequently affected, with the distal interphalangeal joint being the most common amputation level among Long fingers. Multiple digit amputations occurred in 17.5% of cases and predominantly in patients suffering from leisure trauma. This study provides a detailed epidemiological and etiological analysis of digit amputations, revealing a young, male-dominated cohort with a significant proportion of occupational trauma. The findings highlight the need for targeted prevention strategies and informed planning of trauma care resources.

## Introduction

Digit amputations are among the most frequently encountered traumatic injuries of the upper extremity, often resulting from occupational accidents, industrial machinery, or sharp object trauma^1^. These injuries not only lead to significant functional impairment, particularly in hand dexterity and grip strength, but also pose substantial psychological and socio-economic challenges for affected individuals^2–6^. Since hand function is essential for many different work and leisure activities, its preservation is of utmost importance in the treatment of digit amputations. Since replantation is not always indicated, feasible, or successful, the appropriate management of definitive amputations remains a critical aspect of care for healthcare professionals involved in the treatment of these injuries.

Understanding the epidemiology and etiology of these injuries can help in their immediate and long-term treatment as well as in their prevention. Different epidemiological studies have indicated that males, particularly those in older age groups, are disproportionately affected^7–9^. The unique anatomical and functional importance of different amputation levels and affected digits influences both the surgical treatment approach and possible outcomes^4^. The thumb, index and middle finger, for example, are crucial for grip and precision, making injuries to these digits particularly debilitating. Replantation or reconstructive techniques are often prioritized in cases where a definitive amputation could lead to functional deficits^10^. However, unsuccessful primary replantations can lead to surgical revision, possibly resulting in a secondary amputation of the affected digit.

The temporal distribution of digit injuries—which potentially mirrors patterns observed in peripheral nerve trauma—may offer valuable insights for optimizing preventive measures and guiding resource allocation within trauma care systems^11^. Seasonal trends may correlate with increased outdoor activities, construction projects, and agricultural work. Additionally, variations in injury incidence across different days of the week may reflect fluctuations in occupational exposure and recreational activity, further informing targeted prevention and planning efforts. The impact of such patterns on emergency care systems underscores the need for resource allocation and preventive strategies.

The present study aims to add to the existing body of knowledge by providing comprehensive data on more than 2,000 digit amputations over a 16-year period at a single institution. By analyzing demographic and injury factors, including injury mechanisms and anatomical distributions, this study aims to identify key determinants of injury risk and surgical outcomes.

## Results

**Fig. 1:**
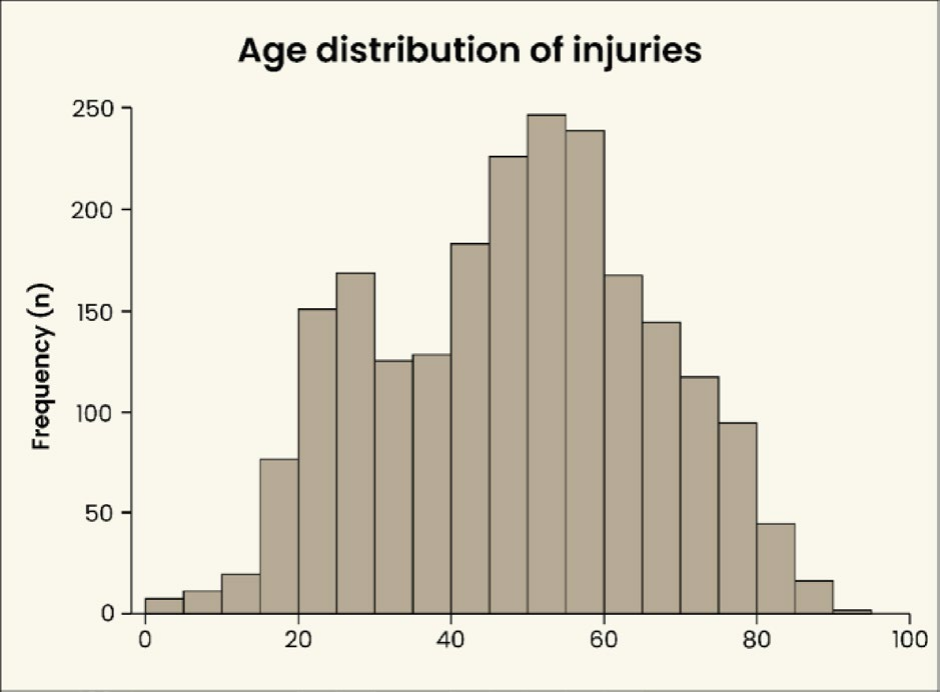
Histogram of age distribution of digit amputations. Bars represent 5-year-steps. Two peaks are seen: at the age of 20–30 years and 40–60 years.

A total of 1768 patients with 2179 digit amputations were included in the final analysis. The majority of the amputations (*n* = 1,941; 89.1%) occurred in male patients. The median age at the time of injury was 49 years (IQR: 34–61 years), with two peaks visible in the age distribution, between the ages of 20 and 30 years as well as between 40 and 60 years (Fig. 1). Basic epidemiological characteristics are shown in Table 1.

**Table 1:** Basic epidemiological characteristics of the cohort.

Variable	*n* = 2,*179*
Male sex, *n (%)*	1,941 (89.1%)
Age, *median (IQR)*,* years*	49 (34–61)
**Insurance status**	
Public, *n(%)*	1,123 (51.5%)
Occupational, *n(%)*	837 (38.4%)
Private, *n(%)*	127 (5.8%)
Occupational trauma, *n (%)*	843 (38.7%)
Right hand affected, *n(%)*	1,042 (47.8%)
**Mechanism of amputation**	
Sharp trauma, *n(%)*	1101 (50.5%)
Blunt trauma, *n(%)*	723 (33.2%)
Avulsion, *n(%)*	216 (9.9%)
Burn/explosion injury, *n(%)*	68 (3.1%)

**Fig. 2:**
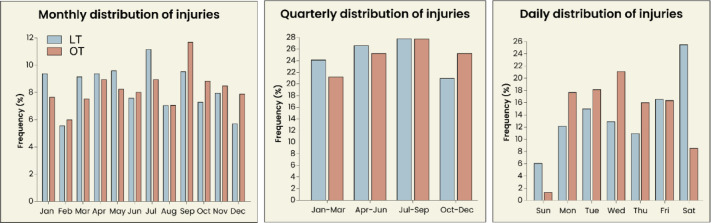
Temporal analysis of digit amputations. Injury frequency is sorted by monthly, quarterly and daily number of cases. Rates of occupational and leisure trauma are shown separately. The highest monthly occurrence was seen in July for leisure trauma and in September for occupational trauma. This is also reflected in the increased frequency reported in the third quarter of the year. When sorted by day of the week, Friday and Saturday had the highest case numbers for leisure trauma, and Tuesday and Wednesday for occupational trauma. LT = leisure trauma, OT = occupational trauma.

When combining both leisure-related and occupational injuries, the total number of amputations was highest in September (*n* = 227; 10.4%), followed by July (*n* = 226; 10.4%) and April (*n* = 202; 9.3%). The second and third quarters of the year showed the highest average incidence of amputations, with 26.2% (*n* = 571) and 27.9% (*n* = 608), respectively. Most amputations occurred on Saturdays (*n* = 415; 19.5%), followed by Fridays (*n* = 362; 16.6%) and Tuesdays (*n* = 356; 16.3%). The lowest incidence was on Sundays (*n* = 95; 4.4%). Temporal distributions are visualized in Fig. 2.

More than a third of amputations (*n* = 843; 38.7%) were occupational, with occupational amputations significantly (*p* < 0.001) more likely to occur during workdays (Monday to Friday) than on the weekend. Amputations in patients younger than 40 years old were significantly (*p* < 0.001) more likely to be the result of an occupational injury. When focusing on patients in the standard working age (18–65 years), patients between the ages of 18–40 years showed significantly increased rates of occupational amputations compared to patients aged 40–65 years (54.3% vs. 42.9%; *p* < 0.001). Men were also significantly more likely to suffer a digit amputation at work than women (40.1% vs. 26.9%; *p* < 0.001). 51.5% (*n* = 1,123) of amputations were covered by public insurance, 38.4% (*n* = 837) were covered by occupational insurance, and 5.8% (*n* = 127) by private insurance.

The majority of amputations (*n* = 1101; 50.5%) were due to sharp injuries, followed by blunt trauma (*n* = 723; 33.2%), avulsion injuries (*n* = 216; 9.9%), and burn or explosion injuries (*n* = 68; 3.1%). In occupational amputations, the predominant injury mechanisms were blunt injuries (*n* = 438, 51.96%), followed by sharp injuries (*n* = 268; 31.97%), avulsion injuries (*n* = 108; 12.8%) and burn or explosion injuries (*n* = 2; 0.2%). Amputations resulting from leisure activities were primarily caused by sharp injuries (*n* = 833; 62.4%), followed by blunt trauma (*n* = 285; 21.33%), avulsion injuries (*n* = 108; 8.1%) and burn or explosion injuries (*n* = 66; 4.9%).

1,137 (52.2%) of amputations occurred on the left hand and 1,042 (47.8%) on the right hand. The index finger was the most frequently affected (*n* = 615; 28.2%), followed by the middle finger (*n* = 507; 23.3%), the ring finger (*n* = 416; 19.1%) and the thumb (*n* = 327; 15.0%). The little finger was the least frequently affected (*n* = 308; 14.1%).

**Fig. 3:**
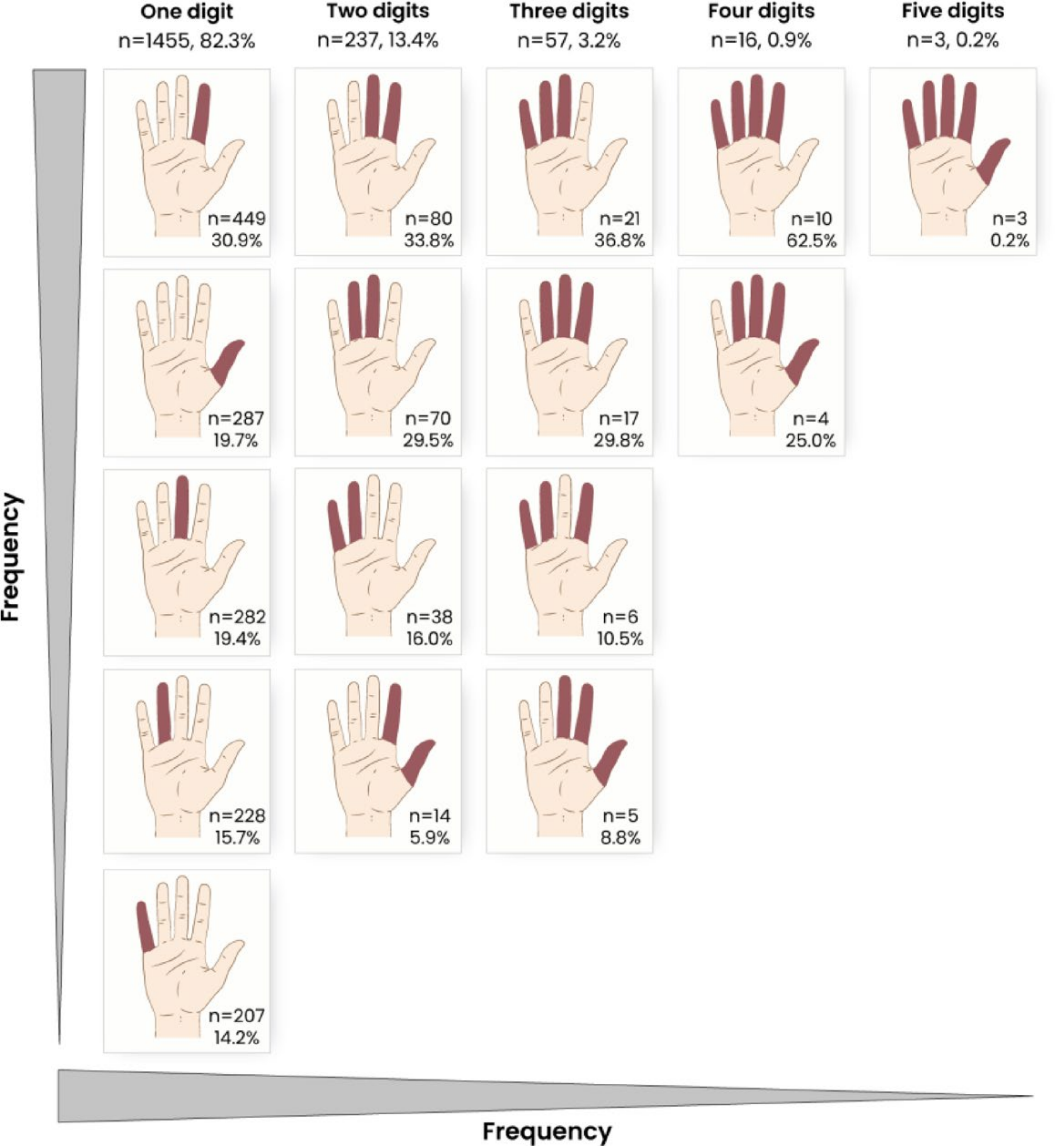
Common digit combinations in multi-digit amputations. Percentages are based on the total cases per column; for example, percentage of little finger amputations from the total of one-digit amputations.

1454 (82.2%) patients had a single-digit amputation, 238 (13.5%) patients a two-digit, 57 (3.2%) patients a three-digit, 17 (1.0%) patients a four-digit, and two (0.1%) patients a five-digit amputation. In patients with occupational trauma (*n* = 245; 29.1%), multi-digit amputations occurred significantly less frequently (*p* = 0.001) than in patients with leisure trauma (*n* = 480; 35.9%). In single-digit amputations, the most frequently affected digit was the index finger (*n* = 449; 30.9%), followed by the thumb (*n* = 287; 19.7%), the middle finger (*n* = 282; 19.4%), the ring finger (*n* = 228; 15.7%) and the little finger (*n* = 207; 14.2%). In two-digit amputations, the combination of the index and middle finger occurred most frequently, making up 33.6% (*n* = 80) of two-digit amputations, followed by the middle and ring finger with 29.4% (*n* = 70) and the fourth and fifth finger with 16.0% (*n* = 38). An amputation of the thumb and the index finger was seen in 5.9% (*n* = 14) of two-digit amputations. In three-digit amputations, the most frequent combinations were third, fourth and fifth finger amputations (*n* = 21; 36.8%), followed by second, third and fourth finger amputations (*n* = 17; 29.8%) and second, fourth and fifth finger amputations (*n* = 6; 10.5%). Concurrent amputation of thumb, index and middle finger occurred in 8.8% (*n* = 5) of three-digit cases. In four-digit amputations, the combination of second to fifth digit made up 58.8% (*n* = 10) of cases, followed by the amputation from thumb to ring finger in 23.5% (*n* = 4) of cases. Common combinations in multi- digit amputations are shown in Fig. 3.

In long finger injuries, the most frequent amputation level was the distal interphalangeal joint (DIP) in 33.5% (*n* = 575) of cases, followed by the middle phalanx and the distal phalanx with 19.3% (*n* = 331) and 16.9% (*n* = 290), respectively. An amputation at the proximal phalanx was seen in 11.4% (*n* = 195) of cases and at the proximal interphalangeal joint in 11.5% (*n* = 198) of cases. A residual length limited to the metacarpophalangeal joint (MCP) occurred in 5.5% (*n* = 95) of cases. In 0.7% of cases the amputation occurred through the metacarpal bone (*n* = 12), and in 1.1% (*n* = 19) of cases, a total metacarpal ray amputation through the carpometacarpal joint was performed.

In thumb amputations, the most frequent amputation level was the distal phalanx in 53.6% of cases (*n* = 155), followed by the interphalangeal joint (IP) in 33.6% (*n* = 97). An amputation through the proximal phalanx was seen in 10.4% (*n* = 30) of thumb amputations and through the MCP in 1.4% (*n* = 4). 1.0% (*n* = 3) of thumb amputations were through the metacarpal bone.

**Fig. 4:**
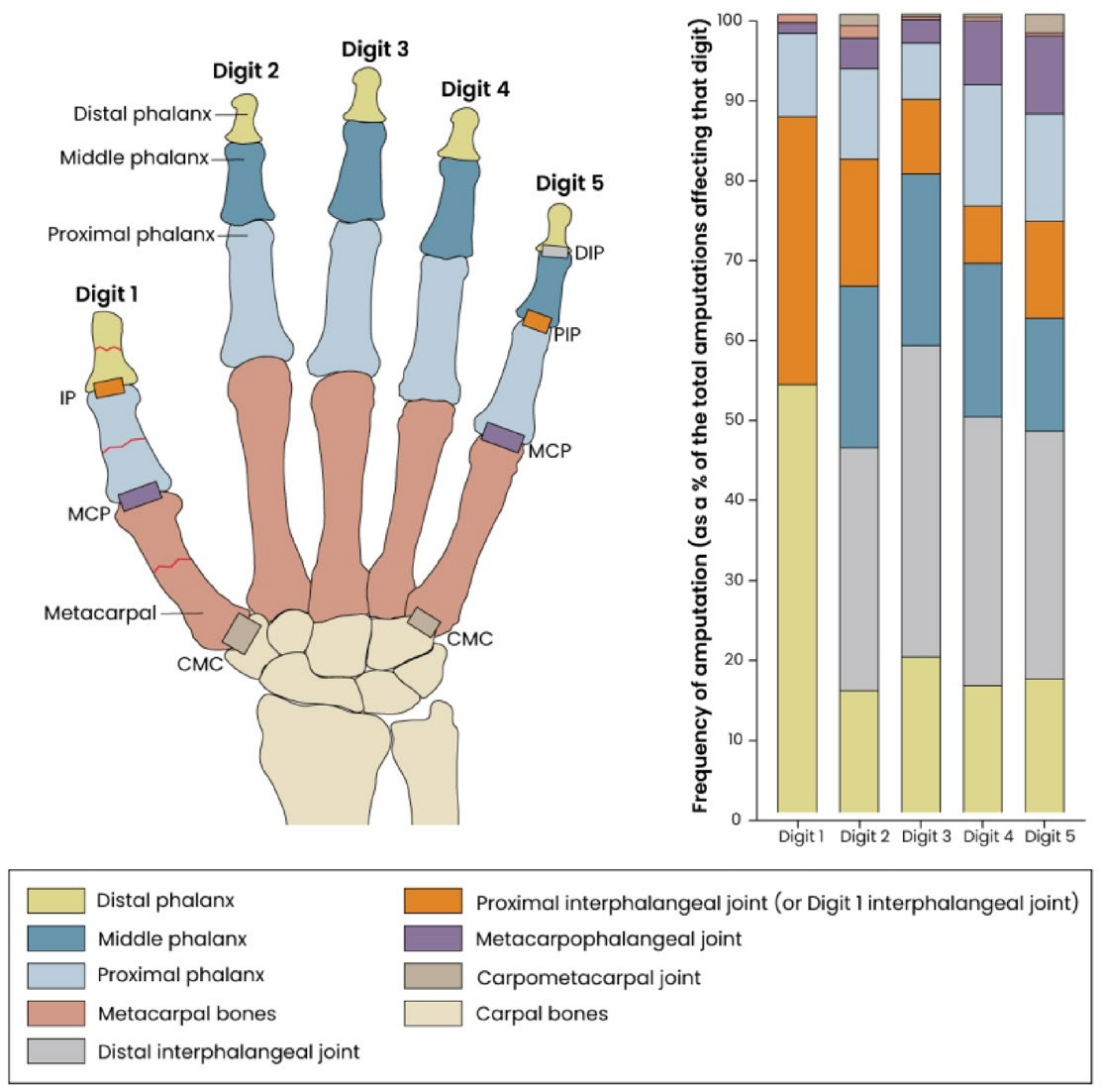
Amputation levels distribution per digit. Shown is the percentage of amputations for different levels, including phalanges, interphalangeal joints and metacarpophalangeal joints, for each digit (D1-D5).

Patients suffering from leisure amputations (*n* = 105; 7.9%) were significantly more frequently (*p* < 0.001) affected by amputations at the MCP or proximally than patients with occupational trauma (*n* = 29; 3.4%). The distributions of amputation levels are visualized in Fig. 4.

The mean surgery time was 48 min (IQR: 29–103 min). Soft tissue coverage was performed using direct wound closure in 40.1% (*n* = 852) of cases. In 36.4% (*n* = 774) of cases, a volar filet flap was used, and in 7.2% (*n* = 152), a dorsal filet flap was used. In 14.4% (*n* = 307) of cases, other regional flaps were used, and in 1.9% (*n* = 41) of cases, other methods for soft tissue coverage were used. This group of other methods included full-thickness skin grafts, groin flaps, and coverage using a semi-occlusive dressing.

## Discussion

The present study provides a comprehensive analysis of over 2,000 digit amputations, describing epidemiological factors, mechanisms of injury, and levels of single- and multi-digit amputations. In this study, we present data collected at a German level I trauma center over the course of 14 years, providing insights into a highly relevant and common aspect of hand surgery. By offering data on a variety of aspects of digit amputation of this large cohort, also comparing leisure and occupational amputations in temporal distributions, injury mechanisms and injury severity, this study can help to improve adequate prevention and treatment.

Our data showed a large male predominance (89.1%) in the study cohort. This is in agreement with prior research, for example, Magnéli et al. found more than three-quarters of a Swedish cohort of partial hand and digit amputees to be male^7^. A bimodal age distribution, peaking at 20–30 and 40–60 years, was also seen in our study cohort. Magnéli et al. identified a correlation between increasing incidence and increasing age, while Reid et al. found a peak in children below the age of five and in adults, again a rising number of digit amputations with increasing age^7,12^. Although our findings do not reflect these results, the difference may be rooted in the fact that more than a third of the amputations in our study were occupational, as our trauma center is a primary center for occupational injuries. Patterns of age distributions similar to those described in our study were also described in other hand injuries, like phalangeal fractures^13^. The first age peak in our study might be attributable to a higher risk among young males to suffer occupational injuries, as described in previous studies^14,15^. This hypothesis is further underscored by the significantly increased ratios of males and patients below the age of 40, who presented with an occupational digit amputation in our study population. This age distribution might reflect a group of workers in early career phases of manual occupations with less experience and high exposure to heavy machinery on one hand and a group of older patients with possibly reduced dexterity, vision, or reaction times and possibly familiarity with equipment leading to decreased caution^16,17^. The drop after the age of 65 may reflect retirement and reduced occupational exposure. This highlights the importance of age-specific safety interventions. Younger, less experienced individuals, as well as older workers, should undergo targeted training to prevent these severe injuries.

The temporal distribution of digit amputations found in our study shows the highest rate of amputations on Saturdays, Fridays, and Wednesdays. While occupational amputations were significantly more likely to occur during weekdays, the high occurrence of amputations on Saturdays further supports the hypothesis of inadequate use of protective equipment during leisure activities, potentially leading to amputations. Most amputations occurred in the third quarter (July-September), which might be due to a high frequency of outdoor activities involving machinery, like saws, putting inadequately trained and equipped individuals at risk. The low incidence in August could be attributed to school holidays and people traveling abroad during this month. These findings could serve to improve care during these seasons, exemplarily through adequate staffing in trauma centers, and to plan targeted prevention in earlier months through public health and safety campaigns.

The mechanisms of injury in our cohort were predominantly sharp trauma in more than a half of all cases, followed by blunt injuries and avulsions. Renfro et al. found power saws, and Long et al. machinery in general to be the most frequent cause of digit amputations^9,18^. In our cohort, the predominant mechanism of occupational injuries was blunt trauma, while leisure amputations were predominantly caused by sharp trauma. This might reflect the adequate use of safety equipment like protective gloves in workplaces, which prevent sharp injuries like cuts, but not blunt trauma like crush injuries from heavy machinery. During leisure activities, this type of safety equipment might be used less frequently, leading to a higher number of preventable sharp injuries. This should be addressed through safety and awareness campaigns targeted to increase the use of protective equipment during high-risk activities, like the use of saws, at home. Although the literature does not describe the injury mechanisms in a homogenous way, this distribution reflects common workplace and recreational hazards. While sharp injuries in our study also included injuries resulting from power saw accidents, future studies should differentiate the etiology in a more detailed way by looking at both, the injury type, including sharp, blunt, and others, as well as the method of injury differentiating between frequently responsible tools like knives, saws and other machinery. Avulsion and crush injuries, which often involve extensive tissue damage, pose significant reconstructive challenges, while sharp injuries are typically associated with better overall outcomes^19^.

The index finger was most frequently affected in single-digit amputations, followed by the thumb and middle finger. This is highly relevant, as these digits play a key role in hand function, regarding both precision and strength. This, on the other hand, might also be the reason for their frequent involvement in amputation injuries. Therefore, protective equipment should specifically address these digits. The distribution of amputation levels roughly shows an increasing rate of amputations from proximal to distal, but the DIP is more frequently affected than the distal phalanx in all digits except the thumb. The distribution of amputations among digits and levels is overall similar to the findings of Sagiv et al.^4^ Distal amputations, while less debilitating than proximal amputations, can still result in functional deficits, especially for tasks requiring fine motor control. Conversely, proximal amputations, can disrupt hand biomechanics, significantly affecting grip stability and range of motion, leading to lower sensory and motor function outcomes^4^. Even if proximally amputated digits are successfully replanted, they are still associated with poorer function compared to more distal amputations following replantation^5^. Amputations at the MCP level or proximally were significantly more frequent in patients suffering from leisure amputations. This might be rooted in inappropriate handling of tools, potentially leading to high-energy mechanisms like crush injuries, frequently affecting proximal sections of the digit or the inadequate use of personal protective equipment.

Multi-digit amputations represent a substantial clinical challenge, exponentially increasing functional impairment^3,4,20^. In our study, 13.4% of patients experienced two-digit amputations, with the combined loss of the index and middle fingers being the most common injury (33.6%). The loss of these digits can severely compromise precision gripping, especially when the index and middle finger are amputated. And when the ring or little finger is amputated, this can lead to a decrease in power grip strength. Previous studies have shown that patients with multi-digit amputations often regain only about half of their preinjury grip strength^3^. These functional consequences can significantly impair activities of daily living and occupational tasks^3,20^. Even more complex are cases involving three or more digits, which were seen in 3.2% of patients. The most frequent combination, the middle, ring, and little finger can result in profound deficits, as the remaining digits are likely insufficient for functional compensation^3^. Although the loss of one or more Long fingers can be functionally compensated, this is not adequately the case for thumb amputations, which represented 15% of our cohort. The thumb and its opposition ability significantly contribute to hand function, these amputations can, therefore, be especially debilitating in terms of the overall hand functional level, especially at a proximal level^3,10,21^. The simultaneous amputation of multiple digits, even if this does not include the thumb, leads to significantly worse functional outcomes compared to single-digit amputations^3,4^. This, in the long term, leads to socio-economic consequences, including rising direct and indirect costs^22^. Multi-digit amputations were significantly more frequent in patients suffering from leisure trauma, which again might be rooted in inappropriate risk assessment and the inadequate use of personal protective equipment. The comparatively higher number of proximal and multi-digit amputations in leisure patients underscores the importance of hazard awareness and adequate use of machinery with the appropriate protective equipment, which should be addressed by educational campaigns.

The replantation–amputation sequence reflects the clinical challenge of identifying which digits are salvageable at the time of injury. Different factors have been associated with higher survival rates in digit replantations, including clean-cut injury mechanisms, the absence of smoking history, and the ability to complete arterial and venous repairs without the need for vein grafts^23^. Proper preservation of the amputated digit markedly improves the likelihood of a successful replantation attempt, with evidence supporting immediate placement of the amputate in cold ischemia after injury^23^. Failed replantation attempts can lead to prolonged recovery, increased healthcare utilization, and ultimately suboptimal functional outcomes^24,25^. Accurate early decision-making is therefore essential to avoid unnecessary procedures. Tools such as the recently published Mangled Digit Severity Score offer a structured approach to support these decisions by estimating the likelihood of functional salvage^26^. While large-scale validation is currently pending, incorporating such predictive tools into clinical workflows may help improve outcomes and guide resource allocation in complex digital trauma^26^.

Regardless of their functional consequence, digit amputations can also lead to long-term sequelae through chronic pain. Vlot et al. described that 6.6% of patients developed symptomatic neuroma following digit amputation, leading to pain and in two thirds of the study population to operative revision^27^. de Lange et al. found 18% of digit amputation patients to have neuropathic pain^28^. The high prevalence of this potentially debilitating pain form underscores the importance of its prevention. Although different methods like end-to-end neurorrhaphy or Targeted Muscle Reinnervation (TMR) have been described to prevent or treat symptomatic neuromas distal to the wrist, there is currently no consensus on the optimal method^29–32^.

Following amputation, rehabilitation is critical, especially for patients with multi-finger or thumb amputations, where functional recovery is complex^4^. A tailored approach that incorporates physical and occupational therapy, as well as adaptive tools or prosthetics, is essential^33^. Regeneration of overall hand function improves with time after the amputation, underscoring the importance of continuous training^3^. Prosthetics and advanced reconstructive procedures like toe transfers offer the possibility to regain function after amputations, especially following thumb amputations with their functional impact^34–38^. With recent developments in the field of amputation and prosthetics, improved outcomes might become available to patients with digit amputation in the future^36–38^. In particular, for patients with thumb amputations, innovative prosthetic reconstruction models that integrate diverse materials and techniques hold promise for enhancing function and quality of life^36–38^. While future research should investigate ways to further enhance hand function in patients with digit amputations, equal attention should be given to implementing targeted safety strategies aimed at preventing such injuries in the first place.

This study presents a detailed analysis of epidemiological and etiological factors of over 2,000 digit amputations. While these findings can help hand surgeons better understand the challenges arising from digit amputations, this study also has limitations. Due to its retrospective nature, long-term functional outcomes of the described digit amputations are not included in this study. Further, this study focuses deliberately on amputations without a replantation attempt or with failed replantation. However, successful replantations should always be considered in the management of digit amputations, especially in amputations with high functional impact.

## Conclusion

This study provides a comprehensive analysis of 2,179 digit amputations treated at a German Level I trauma center, offering valuable insight into the epidemiology and etiology of these injuries. The findings highlight a predominantly young and male patient population presenting with acute, functionally significant amputations involving one or more digits. By characterizing this demographic and injury pattern, the data can support hand surgeons in optimizing initial management and anticipating reconstructive needs. Future research should focus on long-term functional outcomes and aim to refine both surgical techniques and rehabilitative strategies to enhance patient quality of life.

## Methods

All patient records from a German level I trauma center were screened retrospectively between January 2008 and December 2021 to identify all cases of digit amputations using the digital hospital information system. Ethical approval and a waiver of informed consent were obtained from the local ethics committee (Ethics Committee of the State Medical Association of Rhineland-Palatinate, Mainz, Germany, reference number 2021–16091). The study was carried out in accordance with all relevant guidelines and regulations.

All patients with digit amputations were eligible for inclusion. Cases in which a successful replantation was performed were not included. Data extraction was performed by two independent reviewers in a pseudonymized manner using Microsoft Excel Version 16.90.2 (Microsoft Corporation, Redmond, WA, USA). An anonymized database was then created. STATA Version 17.0 (StataCorp LLC, College Station, Texas, USA) was used for analysis, and GraphPad PRISM Version 10.1.1 (GraphPad Software, Boston, MA, USA) was used for visualization. For the analysis of the amputation level, the final intraoperative amputation level was used.

Categorical variables are shown as frequencies (n) and percentages (%) and were analyzed using the chi-square test. Continuous variables were assessed for normality using the Shapiro-Wilk test. Normally distributed variables are shown as mean and standard deviation and were analyzed using the double-sided t-test, while non-normally distributed variables are shown as median and interquartile range and were analyzed using the Mann-Whitney U test. P-values < 0.05 were considered statistically significant.

## Data Availability

The datasets generated and/or analysed during the current study are not publicly available due to patient privacy and data protection regulations, but are available from the corresponding author on reasonable request and with appropriate institutional approvals.
